# The critical role of phase difference in theta oscillation between bilateral parietal cortices for visuospatial working memory

**DOI:** 10.1038/s41598-017-18449-w

**Published:** 2018-01-10

**Authors:** Philip Tseng, Kai-Chi Iu, Chi-Hung Juan

**Affiliations:** 1 Graduate Institute of Humanities in Medicine, Taipei Medical University, Taipei City, Taiwan; 2 TMU – Research Center of Brain and Consciousness, Taipei Medical University, Taipei City, Taiwan; 3 Shuang-Ho Hospital, Taipei Medical University, New Taipei City, Taiwan; 4 Institute of Cognitive Neuroscience, National Central University, Taoyuan City, Taiwan

## Abstract

Visual working memory (VWM) refers to people’s ability to maintain and manipulate visual information on line. Its capacity varies between individuals, and neuroimaging studies have suggested a link between one’s VWM capacity and theta power in the parietal cortex. However, it is unclear how the parietal cortices communicate with each other in order to support VWM processing. In two experiments we employed transcranial alternate current stimulation (tACS) to use frequency-specific (6 Hz) alternating current to modulate theta oscillation between the left and right parietal cortex with either in-phase (0° difference, Exp 1), anti-phase (180° difference, Exp 2), or sham sinusoidal current stimulation. In Experiment 1, in-phase theta tACS induced an improved VWM performance, but only in low-performers, whereas high-performers suffered a marginally-significant VWM impairment. In Experiment 2, anti-phase theta tACS did not help the low-performers, but significantly impaired high-performers’ VWM capacity. These results not only provide causal evidence for theta oscillation in VWM processing, they also highlight the intricate interaction between tACS and individual differences—where the same protocol that enhances low-performers’ VWM can backfire for the high-performers. We propose that signal complexity via coherent timing and phase synchronization within the bilateral parietal network is crucial for successful VWM functioning.

## Introduction

Visual working memory (VWM) refers to the complex interaction between visual attention and short-term memory, and is essential for maintaining a stable perception of the world by preserving information across blinks and saccades^1^. It has been shown that he capacity of VWM is positively correlated with fluid intelligence^2,3^, and decades of psychological testing using a change detection task have suggested that people’s VWM capacity averages around 3 to 4 simple features in a brief timeframe^4^.

### Parietal Cortex and VWM

In the context of visuospatial memory where people have to remember where things are, neuroimaging studies have found a network of brain regions that support the integrity of VWM. These areas include the occipital cortex^5^, the prefrontal cortex^6^, and the posterior parietal cortex (PPC)^7,8^. Specifically, PPC seems to be a major reason why people sometimes cannot detect seemingly obvious changes across blinks, saccades, or visual disruptions^9^. In a seminal study by Beck and colleagues^10^, the authors compared BOLD responses between detection trials and miss trials and found greater contrast in bilateral PPC. Similarly, Todd and Marois^7^ also found that people’s bilateral PPC activity is positively correlated with their VWM load, until it eventually plateaued at 3 and 4 items. Similar correlations was also observed using EEG, where amplitude from parietal areas can be predictive of good versus poor VWM performance^11^.

Causal evidence between PPC and VWM performance came from a number of noninvasive brain stimulation studies. Beck *et al*.^12^ used transcranial magnetic stimulation (TMS) to disrupt bilateral PPC activity and found that right PPC disruption yielded the strongest impairment to VWM performance. Subsequently, in previous study^13^, it demonstrated that such TMS disruption was most effective early, during the encoding and maintenance stage of VWM processing. Using transcranial direct current stimulation (tDCS), a technique that uses electric current to induce excitatory or inhibitory reactions from the brain, researchers have also found that anodal tDCS over the right PPC can effectively boost people’s VWM capacity and performance^14,15^. This boost, however, did not work for everyone. In two studies^14,16^, they reported that only participants below average were able to benefit from anodal tDCS over the right PPC, with relevant EEG markers that corresponded with the behavioral data. Specifically, Hsu *et al*.^16^ found that increased alpha power seemed to be detrimental to participants’ VWM performance, and the effect of tDCS was, in part, successful because it brought low-performers’ alpha power down near the stimulated parietal sites. On the other hand, the high-performers did not show an improvement or impairment after stimulation. Together, these studies suggest that signals of certain frequency bands can be indicative of people’s VWM performance, and that brain stimulation can be a useful approach in inducing such oscillation to establish causal evidence and neural entrainment, as long as the interactive effect between brain stimulation and intrinsic individual differences (e.g., low- vs. high-performers)^17^ is taken into account.

### Theta Oscillation and VWM

Aside from the detrimental effect of occipitoparietal alpha frequency, are there other frequency bands that are important for the processes behind VWM? Animal recording and human EEG studies have implicated an important role for theta and gamma oscillations in WM in general^18^. For example, Canolty *et al*.^19^ found that gamma power is phase-locked to theta oscillation in EEG signals during a WM task. Axmacher *et al*.^20^ have also demonstrated a slowing of theta frequency into which gamma frequency is nested as a function of WM representation in humans. These findings have led Lisman and Jensen^18^ to propose that WM capacity may be tightly linked to the number of gamma cycles that are enclosed within a particular phase of a theta cycle. Importantly, theta oscillation seems to be specifically relevant to VWM processing as it has been linked with better visuospatial WM processing, especially near the parietal region^21^. Using a change detection task, Darriba *et al*.^22^ also observed higher theta power over the parietal-occipital sites when a change was consciously detected.

Recently, one important study by Jaušovec and Jaušovec^23^ tested whether theta oscillation is critical to VWM using transcranial alternating current stimulation (tACS), a variation of electrical stimulation that uses AC current for inducing neural entrainment in the desired frequency^24^ or spike-timing dependent plasticity^25^. These authors applied 6 Hz tACS, with 0° relative phase difference, either over the left frontal (F3) or left parietal (P3) cortex. VWM performance was measured with a standard change detection task^6,26^, and each participant’s memory capacity was estimated with Pashler’s K^27^. Jaušovec and Jaušovec^23^ observed higher VWM capacity and faster P300 latency only when tACS was applied over the left parietal cortex, and not over the left frontal cortex. These important findings demonstrate that in-phase theta tACS over the left parietal cortex can readily improve people’s VWM performance, implying an important role for theta oscillation at the left parietal region.

### Phase and Timing in VWM and tACS

To clarify the function of theta oscillation within the parietal region, one important aspect to probe is the timing or phase relationship during which theta oscillations occur. Observations like these have been reported in a series of studies by Desimone and colleagues^28–30^. For example, in the context of a visual detection task, Gregoriou *et al*.^29^ observed a phase difference of 152° that roughly corresponds to a 8~13 ms lag (i.e., high gamma range) between frontal eye fields and V4, which was suggested as the axonal conductance time and synaptic delays between the two areas. In humans, Baldauf and Desimone^30^ also observed gamma synchronization between the inferior frontal junction, and, depending on which stimulus (face or place) the participants were attending to at the time, the fusiform face area or the parahippocampal place area.

With tACS, Polanía *et al*.^31^ applied theta tACS, with either 0° (in-phase) or 180° (anti-phase) relative phase difference, over the left frontal and parietal cortex before participants performed a verbal WM task. These authors found a dissociation between theta phase and participants’ reaction time (RT): in-phase theta tACS speeded up participants’ verbal WM performance, whereas anti-phase theta tACS slowed it down. Therefore, not only is theta oscillation critical to WM performance, the subtle difference in timing that is reflected in stimulation phase difference can also have profound impact on the neural processes behind WM. The significance of phase differences is still currently under investigation. Polanía *et al*.^31^ proposed a synchronization view of the in-phase vs. anti-phase difference as synchronization vs. desynchronization, respectively. However, with the phase relationship between both electrodes being constantly stable, and thus highly coherent at any given moment, such view of synchronized vs. desynchronized neural entrainment is untenable.

Given that neural transmission takes time, Fell and Axmacher^32^ proposed that any direct communication should result in a phase lag of greater than 0°, whereas a perfectly synchronized relationship (in-phase) may imply two brain regions receiving a common input from a third region since there is no room for transmission lag under in-phase stimulation. This view would predict that anti-phase tACS may not necessarily be disruptive to network activities or detrimental to cognitive processing, which was supported by a recent study^33^. By combining tACS and a VWM color-shape feature binding task, these authors applied either in-phase or anti-phase gamma tACS over participants’ left temporal and parietal cortex, and observed a facilitation effect only in the anti-phase condition, and not in the in-phase condition. Note that the anti-phase facilitation effect was only observed in the lower 50% of participants who performed below average to begin with. Therefore, interactions similar to those between tDCS and individual differences may also be present when using tACS. Together, these results again reinforce the idea that phase lag, as well as individual differences, may be important factors to consider when testing multi-region neural oscillations VWM with tACS.

### The Present Study

The present study aims to apply both in-phase and anti-phase theta tACS over bilateral PPC to investigate the role of theta in supporting VWM. The rationale for such design is twofold: First, although theta oscillation has been demonstrated to be critical to VWM processing using tACS by Jaušovec and Jaušovec^23^, their study focused on left PPC alone by placing the reference electrode over the forehead, and thus was investigating a within-region theta effect in the left PPC. Given that fMRI studies often implicate bilateral PPC functional activities during VWM processing, here we aim to test whether theta is a critical component behind such functional connectivity. Second, if such connectivity implies that the left and right PPC are working in conjunction to support VWM processing, then alterations to their phase relationship should have considerable effect, for better or worse, on its functions and effectiveness. To this end, we conducted two separate experiments, one using in-phase theta (6 Hz) tACS between bilateral PPC, and another using anti-phase tACS on a new group of participants, to probe the role of theta and phase difference in bridging the bilateral PPC network activities in the context of VWM.

## Methods

### Participants

Forty-eight participants with normal or corrected-to-normal vision from National Central University participated in this experiment (24 male, 24 female; mean age 23). Participants were randomly assigned to participate in either Experiment 1 (in-phase theta) or Experiment 2 (anti-phase theta), but not both experiments. A within-subject design was used for each stimulation protocol (i.e., in-phase vs. anti-phase). Therefore, all participants came to the lab on 2 separate days, one active and one sham, scheduled at least one week apart and in counterbalanced order. All experimental and tACS procedures were approved by the Institutional Review Board of Chung-Ho Memorial Hospital, Taiwan, and all participants gave written informed consent prior to participation. In accordance with regulations of the Institutional Review Board, the data obtained in this study cannot be shared with other collaborators or researchers without the written re-consent of the participants.

### tACS Protocol

tACS was delivered through a constant current stimulator (DC-STIMULATOR MC, NeuroConn) with two stimulation electrodes (4 × 4 cm^2^) and a reference electrode (5 × 7 cm^2^). All electrodes were covered with sponge coverings and soaked with saline solution. Target electrodes were located at the left PPC (P3) and right PPC (P4). The reference electrode was placed over the lower left cheek rather than cortical areas in order to minimize any unintended cognitive aftereffects^34^. The waveform was sinusoidal at 6 Hz, delivered at an intensity of 1.6 mA (peak to peak) without DC offset^33^. None of the participants reported discomfort or phosphine either during or after the stimulation. Sham tACS stimulation consisted of the same hardware setup, but the stimulation only lasted 30 sec, and was turned off after that. This is a single-blind design such that participants did not know which session of the two was active or sham. In addition, since tACS has been suggested to have weaker and shorter aftereffect than DC stimulation^35^, in this study we adopted an online stimulation design such that participants received active stimulation during the experiment, which lasted roughly 20~24 minutes (for a similar example in gamma frequency, see Tseng *et al*.^33^).

**Figure 1:**
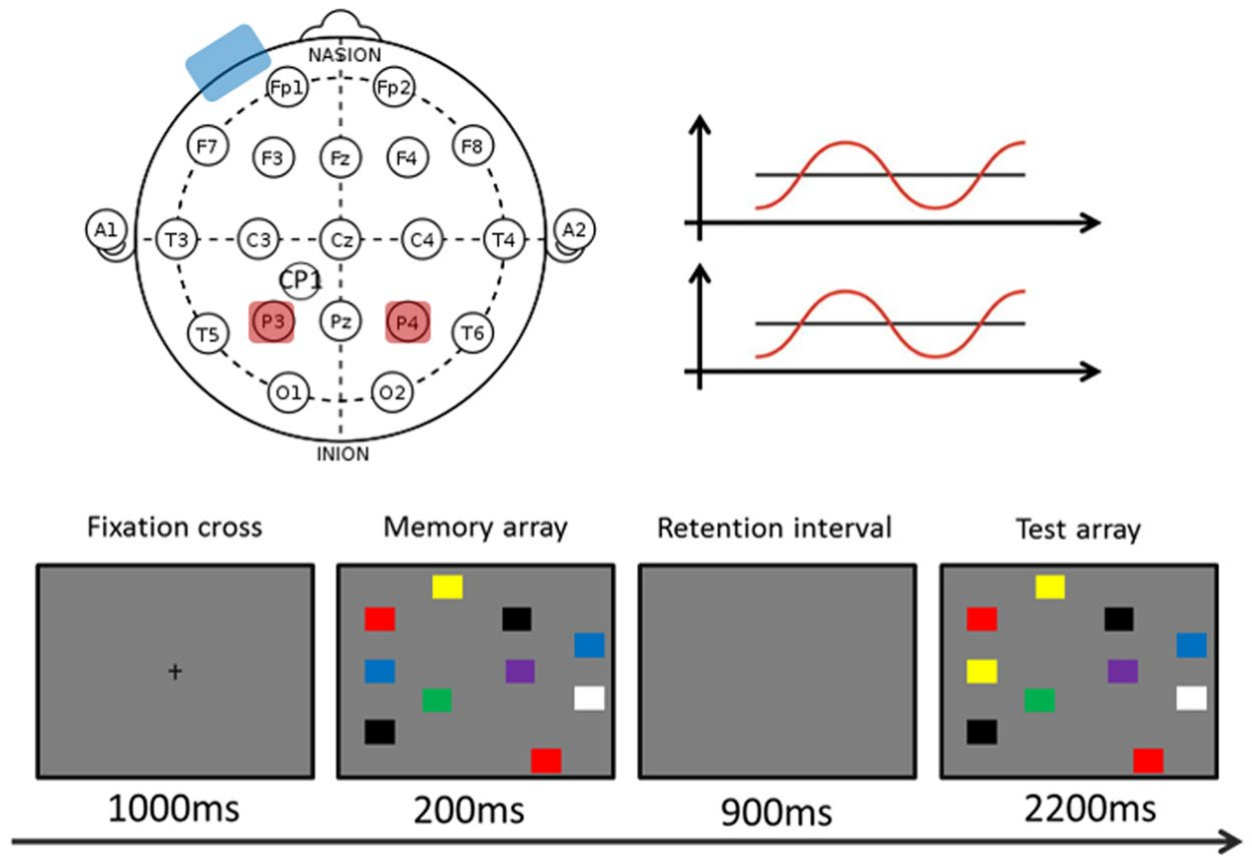
Stimulation sites and trial procedure for Experiment 1: Electrodes were placed over P3 and P4 with no phase offset, and the reference electrode was placed over the left cheek. The trial began with a 1000 ms fixation cross, followed by a memory array of colored squares for 200ms, a retention interval for 900 ms, and a test array for 2200 ms. The test array is different from the memory array 50% of the time.

The 24 participants in Experiment 1 received in-phase theta (0° phase difference between P3 and P4 with reference on left cheek, Fig. 1) on the active tACS day, and sham stimulation on the sham tACS day. The two days were scheduled at least one week apart, and the order of the two were counter balanced across all participants. The same kind of setup was used in Experiment 2, where a new group of 24 participants who are naïve to the purpose of the experiment and have not done any change detection task before, participated in the experiment. They received sham tACS and anti-phase tACS (180° phase difference between P3 and P4) on two different days just like Experiment 1.

### Task and Procedure

Participants sat 57 cm in front of the monitor and performed 3 blocks of 72 trials. Their VWM performance was measured with a change detection task. In this task, participants were to memorize a briefly-presented (1000 ms) sample array that consisted of 10 colored squares. After a short retention interval (900 ms), participants a display of test array and were to indicate whether the test array was identical with the memory array. The entire display extended approximately 31° × 24°. The 10 multi-colored rectangles were 16 mm wide and 13 mm tall. All rectangles were kept at least at a vertical distance of 10 mm and a horizontal distance of 24 mm apart from each other. The color of each rectangle was randomly selected from a pool of 7 highly discriminable colors (red, blue, violet, green, yellow, black, and white), and each color could at most appear in two squares in each array.

This advantage of the change detection task lies in its ability to provide a quantitative estimation of one’s VWM capacity (i.e., how many colored squares of the 10 can be effectively retained). Here we adopted Pashler’s K^27^ formula to estimate each participants’ VWM capacity because (1) Pashler’s K has been suggested to be better suited for whole-display change detection tasks like the ones used here^36^, and (2) to make our study easier to compare with previous VWM tACS study done by Jaušovec & Jaušovec^23^. The Pashler’s K assumes that if one can hold K items in memory from N items of a sample array, then the changed item should be one of the items to be held in memory for K/N items. To avoid biases that may lead to lucky guesses, this procedure takes into account the false alarm rate (F) such that the formula becomes K  =  N times (H − F)/(1 − F).

## Results

### Experiment 1

Participants were sorted based on their natural VWM performance in the sham condition to see whether there was any interaction between individual differences and brain stimulation^17^. This was done because our previous research studies have all suggested that tDCS and tACS are usually more effective for the low performers^14,16,33^, whereas the effect is less pronounced or nonexistent for high performers (for a review, see Juan *et al*.^9^).

Participants were sorted by their median K value (4.097; Hit = 0.53, FA = 0.20) measured in the sham condition (range: 2.202~6.250) and were divided into two groups: high-performers (mean K = 5.405; Hit = 0.60, FA = 0.13) and low-performers (mean K = 3.109; Hit = 0.41, FA = 0.15) based on a median split. A Lilliefors test was conducted to check the assumption of normality in all groups (low-performers sham: p = 0.11; low-performers tACS: p = 0.86; high-performers sham: p = 0.76; high-performers tACS: p = 0.24; all participants sham: p = 0.22; all participants tACS: p = 0.72).

A 2 × 2 mixed-design ANOVA was conducted for the within-subjects factor of tACS (sham vs. in-phase) and between-subjects factor of participant performance (high vs. low). There was no main effect for tACS (F(1,22) = 0.307, p = 0.585), but a significant main effect for performance grouping (F(1,22) = 54.991, p < 0.001) and, most importantly, a significant interaction between tACS and performance grouping (F(1,22) = 9.920, p = 0.005).

**Figure 2:**
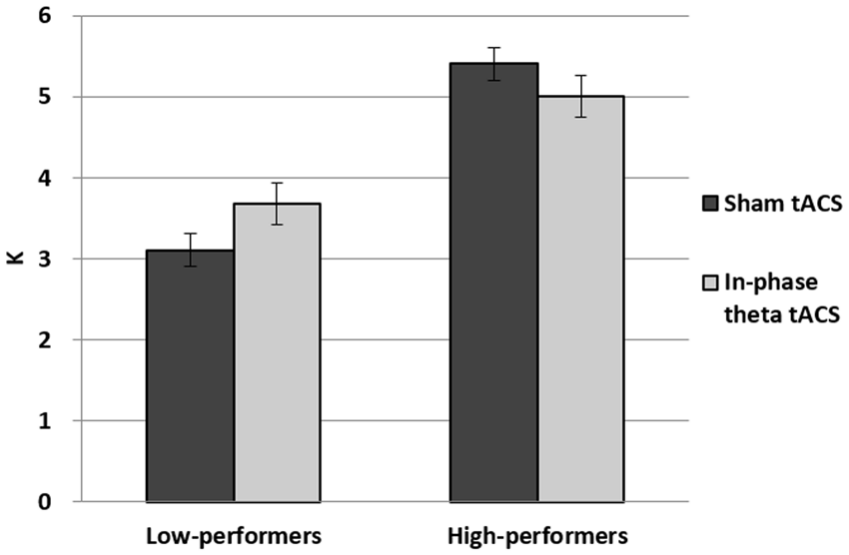
Experiment 1 results: Low-performers showed a significant increase in VWM capacity whereas high-performers showed a marginally significant decrease in VWM capacity.

Subsequent analyses showed that low-performers showed a significant improvement in VWM performance (t(11) = −2.401, p = 0.035) from the sham condition (3.109; std err = 0.2) to the in-phase theta tACS condition (3.673; std err = 0.257) despite counterbalanced sessions across participants. This is consistent with previous research findings. The high-performers, however, exhibited a marginally significant decline in VWM performance (t(11) = 2.039, p = 0.066) from the sham condition (5.405; std err = 0.225) to the in-phase theta tACS condition (5.010; std err = 0.29). Thus, the significant interaction between tACS and innate performance was driven by the tACS effect that goes in opposite directions in low- and high-performers (Fig. 2).

### Experiment 2

Similar to Experiment 1, all 24 participants were sorted by their median K value (4.005; Hit = 0.51, FA = 0.18) from the sham condition (range: 1.667~6.435) and then divided into the high- (mean K = 5.299; Hit = 0.60, FA = 0.15) and low-performing groups (mean K = 3.075; Hit = 0.41, FA = 0.15). Note that the new group of 24 participants also showed similar VWM performance that is on par with participants from Experiment 1, both in low-performers (Exp 1: 3.109; Exp 2: 3.075) and high-performers (Exp 1: 5.405; Exp 2: 5.299), thus providing proper baseline for evaluating the possible effects of tACS. A Lilliefors test was conducted to check the assumption of normality in all groups (low-performers sham: p = 0.03; low-performers tACS: p = 0.80; high-performers sham: p = 0.78; high-performers tACS: p = 0.47; all participants sham: p = 0.44; all participants tACS: p = 0.90).

A 2 × 2 mixed-design ANOVA with the within-subjects factor of tACS (sham vs. anti-phase) and between-subjects factor of participant performance (high vs. low) showed no main effect of tACS (F(1,22) = 2.102, p = 0.161), an expected significant effect of performance grouping (F(1,22) = 32.722, p < 0.001), and lastly, a significant interaction between tACS and performance grouping (F(1,22) = 6.944, p = 0.015).

**Figure 3:**
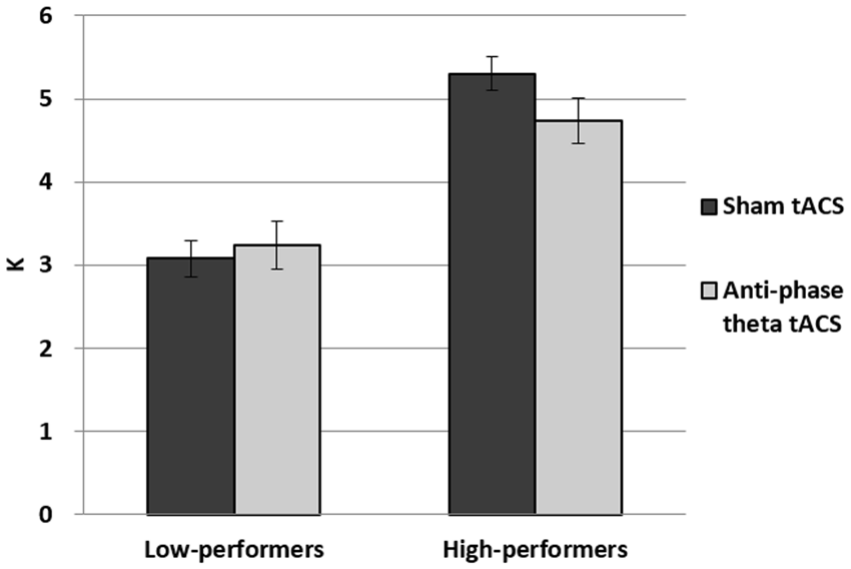
Experiment 2 results: Unlike Experiment 1, low-performers did not show an improvement in VWM performance, whereas high-performers suffered a VWM impairment from anti-phase theta tACS.

Post-hoc comparisons showed that, unlike Experiment 1, low-performers did not show a significant improvement in VWM performance (t(11) = −0.955, p = 0.36; Wilcoxon signed rank test, p = 0.47) from the sham condition (3.075; std err = 0.225) to the anti-phase theta tACS condition (3.237; std err = 0.29). Furthermore, and also unlike Experiment 1, the high-performers here now show a statistically significant decline in VWM performance from the sham condition (5.299; std err = 0.203) to the in-phase theta tACS condition (4.741; std err = 0.271) (t(11) = 2.605, p = 0.024). Therefore, although we have also observed an interaction between tACS and innate performance in Experiment 2, such interaction was driven by a lack of effect in low-performers and a VWM impairment in the high-performers (Fig. 3), a somewhat opposite effect of what we observed in Experiment 1.

## Discussion

The goal of this study was twofold: to test whether theta oscillation is a critical element behind bilateral PPC oscillation, and if so, to probe the appropriate phase difference within the theta frequency on which this parietal network operates. In two experiments, we applied 6 Hz tACS over bilateral PPC with either 0° (Exp 1) or 180° (Exp 2) between left and right PPC. In Experiment 1 (in-phase), active but not sham tACS improved low-performers’ VWM capacity, and in Experiment 2 (anti-phase), active tACS did not affect low-performers’ VWM capacity, but still impaired high-performers’ VWM capacity.

Behaviorally, at first glance the upward trend in low-performers and downward trend in high-performers may look like a regression towards the mean. However, it is important to note that not all protocols (i.e., anti-phase) are able to improve low-performers’ VWM capacity. Indeed, if we look closely at the participants’ performance across the two experiments, participants’ sham condition performances were very consistent in both low- and high-performing groups. Therefore, if the effects of tACS really did originate from a regression toward the mean, then the change in directions of in-phase and anti-phase should be identical, which is not the case in our current results.

### Inter-parietal theta oscillation and VWM processing

The importance of theta oscillation in memory was first demonstrated in the navigation literature, where hippocampal place cells would encode spatial information through phase precession^37^. Later studies have suggested that such increase in theta power was not limited to spatial information, but generalizable to various forms of multi-item WM especially in the encoding and retention timeframe^38,39^. In recent years, the importance of theta oscillation as well as its coupling with other higher frequencies (e.g., alpha, gamma) has been implicated in many types of WM processing^18,40^, sometimes correlating with one’s WM capacity^41^. Notably, this is particularly true for studies that used paradigms requiring the maintenance of precise temporal-order information. For example, using a Sternberg’s task, Jensen and Tesche^42^ observed strong frontal theta activity that correlated with participants’ WM load during the retention period. Similarly, Raghavachari *et al*.^38^ also used a Sternberg’s task and observed increased theta power that increased at the onset of the trial and decreased at the end. Recently, using tACS, Vosskuhl, Huster, and Herrmann^43^ applied anti-phase theta frequency below each participant’s normal task-related theta frequency (i.e., to fit more gamma cycles), and observed improved forward digit-span WM performance. Similarly, Alekseichuk *et al*.^44^ also found that high gamma power (80–100 Hz) over the peak, but not over the trough, of each theta cycle had maximal effect on boosting visuospatial WM, suggesting that a large theta-gamma ratio to be causal to verbal and visual WM processing. Violante *et al*.^45^ also applied in-phase theta tACS over the right frontal and parietal cortex and observed improved 2-back WM performance when cognitive demands were high (e.g., Wu *et al*.^46^), coupled with modulated activity across large-scale networks.

These evidence together have led some to propose that perhaps theta oscillation is involved in the temporal ordering and segmentation of WM information, whereas other high frequencies such as alpha and gamma are involved in distraction inhibition and WM maintenance, respectively^40^.

Despite the evidence above for a role for theta oscillation in WM ordering and segmentation, the temporal order idea seems less applicable to the results of the present study. That is, although the change detection task here also requires WM segmentation between each colored squares to prevent color-location miss-binding errors, there is no segmentation in the temporal domain as in serial presentation like a Sternberg’s task. Thus, the encoding presentation time is too brief (200 ms) or temporally clustered for the temporal order view to explain. As such, an alternative way to explain the current results is that perhaps bilateral PPC theta implicates a “process level” rather than “item level” mechanism^39^. This idea was first proposed by Sauseng and colleagues^39^, where they differentiated between the roles of theta synchronization in maintaining item-specific features (i.e., item level) and in integrating multi-regional brain functions in supporting WM processing (i.e., process level). At the item level, theta power and phase resetting implicates WM formation and retention. Importantly, at the process level, inter-regional theta synchronization implies the involvement of neural transmission across regions to recruit processes such as inhibitory control (e.g., protection from distractors) or other attentional processes (e.g., top-down control) that are vital to, but not (in a strict sense) a part of, WM processing.

This conceptual distinction of item- and process-level theta can also explain some of the different uses of tACS in VWM. Traditionally, tACS has been understood as a tool for multiregional entrainment. However, studies have also used tACS in a non-multiregional way by attaching the return electrode near the orbitofrontal cavity or over the eyebrow^23,47^. Using this setup, and with the target electrode over the left PPC, Jaušovec and Jaušovec^23^ observed improved VWM performance via theta tACS. To compare the K values between the current study and their study, our low-performers showed an improvement from 3.109 to 3.673 in Experiment 1, whereas their participants overall showed an improvement from approximately 3.8 to 4.5 range, which sits right between the mean capacity estimates between our low- and high-performing groups. Although the magnitude of the improvement between the two studies are similar, we have previously interpreted their results as an *intra*-regional, or potentially item-level, effect^33^. And because of such differences in the use of tACS and their respective theoretical implications, we think the current study is best explained at the process level, where theta oscillation as well as its phase synchronization is viewed as an *inter*-regional, rather than *intra*-regional, communication mechanism.

The function of theta oscillation as a long-range connection mechanism has gained support from VWM studies using different techniques. Using EEG, Sauseng and colleagues^48^ observed frontoparietal theta synchronization during VWM encoding processes. Moran *et al*.^49^ also observed similar findings using MEG, where prefrontal and parietal cortices show higher theta and alpha synchronization during VWM encoding period. Recently, Polanía and colleagues^31^ applied in-phase and anti-phase theta tACS (6 Hz) over the left frontal and parietal cortex and observed WM improvement in the in-phase theta condition. The common factor between these studies is that theta synchronization is observed in distal locations, mostly frontoparietal, possibly because theta band is more suitable for long-range transmissions due to its longer wavelength characteristics. Indeed, although we did not investigate the role of frontal cortex in the present study, it is undeniable that the frontal cortex is heavily involved in our change detection task based on the overwhelming support in the literature. This would also explain the puzzling zero phase-lag effect in our data: according to Fell and Axmaher^32^, neural transmissions take time, and a perfect synchronization (i.e., in-phase, 0° difference) between two regions would not be feasible unless they are receiving a common input from a third region. In this light, perhaps the bilateral PPC are functionally connected with the frontal cortices via theta synchronization, and the “driver” of this network will need to be investigated via EEG recording. Nevertheless, as summarized above, we think that there are enough reasons to suspect that theta tACS over a single^23^ or multiple regions^31^ (as in the present study) may both be effective in elevating people’s VWM capacity, but the mechanisms may not necessarily be the same. Here we advocate for a process-level, multiregional synchronization to explain our findings.

### The interaction between tACS, phase variability, and individual differences

In our previous tDCS and tACS studies^14,16,17,33^, we have repeatedly observed a selective VWM improvement in low-performers like the current study, and a lack of effect in high-performers. This homeostatic property of brain stimulation is consistent with previous reports using TMS^50,51^, tDCS^52,53^, and tACS^54^. Note that the impairment effect in Experiment 1 should be interpreted with caution since it is only of marginal significance (p = 0.066). But nevertheless, the anti-phase findings alone would suggest that noninvasive brain stimulation does not work for everyone, and that a more individualized approach to tACS (phase or amplitude) is necessary in order to avoid unanticipated detrimental effects in cognitively functioning, even temporarily^9^.

This finding of VWM impairment in high-performers presents a stark contrast to our earlier tACS experiments^33^ using in-phase and anti-phase gamma tACS over the left parietal and temporal sites: low-performers were able to improve in the anti-phase gamma condition, but no impairment was observed in any condition or any low- or high-performers. To speculate on why this may be the case, there are several differences between the two studies that are potentially responsible, such as the task itself (color-shape binding vs. color-location binding) and the sites of stimulation (left temporopairetal vs. bilateral parietal). However, the stimulation sites and the task procedures from both studies are closely modeled after previous fMRI results, thus there is no *a priori* reason to suspect why a difference in task or brain regions would give rise to such discrepancy in theta and gamma tACS.

One possibility is that perhaps such discrepancy is actually reflecting a difference between the inner workings of theta and gamma synchronization. From the item-level perspective, it has been suggested that long, lower frequency theta cycles can support greater WM capacity due to its capacity to encode more gamma cycles^18^. Accordingly, this would also predict that low-frequency theta oscillation may be less forgiving than gamma oscillation due to the multi-item vs. single-item difference between theta and gamma cycle, respectively, in impacting VWM. In other words, the detrimental effect in theta may be easier to observe given its fewer number of envelopes and higher amount of information at stake within each cycle. Furthermore, even from a process-level point of view, disruption of theta phase synchronization would produce greater impairment in VWM performance due to a disruption in multiregional integration.

Regardless of item- or process-level view of theta, the current findings of a disruptive theta tACS effect in high-performers reinforce the idea that, in optimally-attuned high-performers, any variation of timings in theta frequency can be detrimental to their own endogenous, optimally-tuned, bilateral parietal phase differences. This is based on the assumption that (1) high-performers already possess an optimally amount of variability, or complexity^55^, in their endogenous neural signals, and (2) theta tACS with fixed phase envelope is too monotonous and thus eliminates such intrinsic signal variability. Indeed, it has been reported that high-performers in cognitive tasks tend to exhibit more complex neural signals^56^ (via entropy) than low-performers^57^, and using tACS to boost phase-locking may sometimes be detrimental to WM performance^58^. Looking closer at our results, we speculate that perhaps the optimal phase difference for our task may be somewhere between 0° and 90°, hence why a tACS phase difference of 0° was able to pull low-performers closer to the ballpark and improve their VWM, but a tACS phase of 180° could impair high-performers already-accurate phase relationship more so than the 0° condition. Similar impairment effects have also been reported before and termed as the homeostatic^52,53^ and state-dependent^51^ nature of brain stimulation. Specifically, using TMS combined with a priming paradigm, Silvanto *et al*.^50^ found a negative relationship between participants’ baseline performance and the effect of TMS, where TMS went from facilitatory to inhibitory as people’s baseline performance level increased. Recently, one study by Krause *et al*.^54^ that applied a random noise version of tACS over expert mathematicians has also found an impairment effect of brain stimulation in these high-performing individuals. In contrast, the effect of tDCS and tACS seem to be more beneficial for the low-performers^14,33^, or at least when the task is challenging enough and that the participants still have room for improvement^45,46^. Therefore, the effect of magnetic and electric stimulation seems to be dependent, or interactive, with one’s capability or baseline performance, and therefore should be used cautiously.

Finally, it is important to note that even after in-phase theta tACS, the low-performers are still quite far from the performance level of high-performers. Therefore, tACS-induced variability in phase timing may be quite limited in these low-performers. It is possible that a parametric design of different phase differences may be able to find the specific range of phase offset that is optimal for maximizing the improvement in low-performers’ VWM capacity, which we suspect to be somewhere between 0° and 90° based on the current bidirectional results.

### Limitations

There are several limitations of this study that are worth noting. First, without accurate measurement of current flow, it is difficult to equate the actual effects between the in-phase and anti-phase conditions despite similar setup and identical parameters. Notably, the in-phase setup requires the use of a return electrode, which has been shown to alter current flow^59^ and thus creating unequal amount of current flowing between the conditions. For instance, Saturnino *et al*.^59^ modeled the current flow of the Polania *et al*.^60^ study, which used a fronto-parietal tACS setup with return electrode on the shoulder, and found co-stimulation of orbitofrontal, dorsal and ventral occipital and temporal areas and the cerebellum. In the current study, although we did not use a frontal electrode setup and do not anticipate much frontal co-stimulation, the current nonetheless could have flowed towards the visual and cerebellar area in order to reach the chin reference. Therefore, as with most dual-site tACS results, we recommend caution in interpreting the behavioral effects and more modeling efforts to disambiguate the flow of the electric currents (see Saturnino *et al*.^59^, for an excellent modelling example).

Another limiting factor in the present study is the single-blind design. Even when we attempt to control for the placebo effect with sham sessions, it is difficult to control for the experimenter’s implicit biases that may otherwise influence participants’ attitude towards the tasks. Therefore, a double-blind design would have been better to control for such implicit and explicit biases that can potentially influence the outcome of the study.

Lastly, an alternative explanation to the bilateral PPC entrainment view is that perhaps our results here are driven by one electrode, and not both. In other words, it may be possible that the improvement effect in low performers here is a direct replication of the Jaušovec and Jaušovec^23^ study, with the addition of an impairment effect in high-performers that is coming from right PPC stimulation. Although this speculation is less intuitive in light of the functional connectivity that is often reported between bilateral PPC, it nevertheless is a possibility that we currently cannot rule out. It would be helpful for future studies to test whether tACS entrainment of PPC in hemisphere would create an observable rippling effect on the other hemisphere via fMRI or EEG (for one excellent tDCS-fMRI example, see Ellison *et al*.^61^).

## Conclusion

In summary, the present study demonstrates that theta tACS over bilateral PPC, with no phase-lag between the two electrodes, can improve VWM capacity in low-performing individuals. High-performers, on the other hand, show significant impairment with anti-phase tACS. We propose that the “process level” and “signal variability” frameworks are better suited to explain (1) the role of theta entrainment in our task and (2) the interaction between tACS and individual VWM baselines, respectively. Together, we conclude that theta oscillation between the left and right parietal cortices are causal to visuospatial WM processing.
